# Efficacy and Safety of Programmed Death-Ligand 1 Inhibitor Plus Platinum-Etoposide Chemotherapy in Patients With Extensive-Stage SCLC: A Prospective Observational Study

**DOI:** 10.1016/j.jtocrr.2022.100353

**Published:** 2022-06-08

**Authors:** Kenji Morimoto, Tadaaki Yamada, Takayuki Takeda, Shinsuke Shiotsu, Koji Date, Taishi Harada, Nobuyo Tamiya, Yusuke Chihara, Osamu Hiranuma, Takahiro Yamada, Hibiki Kanda, Takayuki Nakano, Yoshie Morimoto, Masahiro Iwasaku, Shinsaku Tokuda, Koichi Takayama

**Affiliations:** a Department of Pulmonary Medicine, Graduate School of Medical Science, Kyoto Prefectural University of Medicine, Kajii-cho, Kamigyo-ku, Kyoto, Japan; b Department of Respiratory Medicine, Japanese Red Cross Kyoto Daini Hospital, Kyoto, Japan; c Department of Respiratory Medicine, Japanese Red Cross Kyoto Daiichi Hospital, Kyoto, Japan; d Department of Pulmonary Medicine, Kyoto Chubu Medical Center, Kyoto, Japan; e Department of Medical Oncology, Fukuchiyama City Hospital, Kyoto, Japan; f Department of Pulmonary Medicine, Rakuwakai Otowa Hospital, Kyoto, Japan; g Department of Respiratory Medicine, Uji-Tokushukai Medical Center, Kyoto, Japan; h Department of Pulmonary Medicine, Otsu City Hospital, Shiga, Japan; i Department of Pulmonary Medicine, Matsushita Memorial Hospital, Osaka, Japan; j Department of Respiratory Medicine, Omi Medical Center, Shiga, Japan

**Keywords:** Extensive-stage small-cell lung cancer, Elderly, Chemoimmunotherapy, Poor performance status, Cancer cachexia

## Abstract

**Introduction:**

To date, the efficacy and safety of programmed death-ligand 1 (PD-L1) inhibitor plus platinum-etoposide chemotherapy for patients with extensive-stage SCLC (ES-SCLC), with real-world evidence, stratified on the basis of age and performance status (PS), have not been fully investigated. The aim of this study was to evaluate the efficacy and safety of PD-L1 inhibitor plus platinum-etoposide chemotherapy in patients with ES-SCLC.

**Methods:**

This multicenter prospective study evaluated patients with ES-SCLC who received PD-L1 inhibitor plus platinum-etoposide chemotherapy between September 2019 and October 2021.

**Results:**

A total of 45 patients with ES-SCLC received the aforementioned treatment, including 18 elderly (≥75 y old) patients and six patients with a PS of 2. Multivariate analysis indicated that a PS of 2 was a significant independent prognostic factor for progression-free survival and overall survival (*p* = 0.008 and *p* = 0.001, respectively). Of patients with PS of 2 at the initial phase, those that achieved PS improvement during treatment had significantly longer progression-free survival and overall survival than those who did not (*p* = 0.02 and *p* = 0.02, respectively). The incidence of adverse events accompanied with treatment discontinuation was significantly higher in the elderly patients than in the non-elderly patients (*p* = 0.03).

**Conclusions:**

This real-world prospective study found that PD-L1 inhibitor plus platinum-etoposide chemotherapy had limited efficacy in patients with ES-SCLC with a PS of 2, except for cases with improvement of PS during treatment. Owing to the emergence of adverse events and treatment discontinuation, this treatment should be administered with caution in elderly patients with ES-SCLC.

## Introduction

Globally, lung cancer is the leading cause of death in malignant tumors.^1^ SCLC accounts for approximately 15% of all lung cancer cases.^2^ Of which, extensive-stage SCLC (ES-SCLC) is generally considered incurable and accounts for approximately 70% of SCLC cases.^3^ Although ES-SCLC is sensitive to first-line treatment with cytotoxic agents, most patients with ES-SCLC experience recurrence and die within two years from the initial diagnosis.^4^ Recent years have witnessed the era of immune checkpoint inhibitors (ICIs), and chemoimmunotherapy was found to have favorable clinical outcomes for the treatment of patients with lung cancer.^5^, ^6^, ^7^, ^8^ Nevertheless, elderly (≥75 y old) patients with NSCLC tended to have higher rates of treatment discontinuation owing to adverse events (AEs) and severe AEs, including pneumonitis.^9^^,^^10^

Currently, programmed death-ligand 1 (PD-L1) inhibitor plus platinum-etoposide chemotherapy has been recognized as a standard treatment for patients with ES-SCLC in a first-line setting.^7^^,^^8^ To date, there are no reports evaluating the impact of age on the effectiveness of chemoimmunotherapy in patients with ES-SCLC. Therefore, the efficacy and safety of PD-L1 inhibitor plus platinum-etoposide chemotherapy in this population have not been fully elucidated. In addition, previous observational studies revealed that 15% to 30% of patients with ES-SCLC with ECOG-PS of 2, which were ineligible for clinical trials, received systemic chemotherapy as the frontline treatment.^11^, ^12^, ^13^

In this observational study, we investigated the safety and efficacy of PD-L1 inhibitor plus platinum-etoposide chemotherapy in patients with ES-SCLC, including vulnerable populations.

## Materials and Methods

### Patients

Between September 2019 and October 2021, we prospectively enrolled consecutive patients diagnosed with having ES-SCLC who received PD-L1 inhibitor plus platinum-etoposide chemotherapy at the 10 included institutions in Japan. The primary objective of this analysis was to elucidate the association between patient characteristics and progression-free survival (PFS) in this cohort. The data cutoff date was December 31, 2021. The decision to administer PD-L1 inhibitor plus platinum-etoposide chemotherapy and to evaluate its efficacy and toxicity were based on the investigator's discretion. The dose reduction criteria were not specified. All patients were followed-up every 8 to 12 weeks to evaluate their response to treatment using conventional computed tomography scanning and magnetic resonance imaging. Tumor response was determined on the basis of the Response Evaluation Criteria in Solid Tumors guidelines, version 1.1.

PFS was defined as the time from the initiation of PD-L1 inhibitor plus platinum-etoposide chemotherapy to the date of disease progression or any cause of death. Overall survival (OS) was defined as the time from the first administration of PD-L1 inhibitor plus platinum-etoposide chemotherapy to any cause of death.

The inclusion criteria of this study were as follows: (1) patients aged 20 years or older; (2) patients with untreated advanced SCLC, with a pathologic diagnosis of SCLC confirmed by investigating the tumor tissue specimen; (3) ECOG-PS: 0 to 2; and (4) patients with assessable lesions on the basis of the Response Evaluation Criteria in Solid Tumors guidelines (version 1.1). The exclusion criteria were as follows: (1) patients who were deemed inappropriate by the physician in charge of the study and (2) patients for whom evaluation using residual specimens after pathologic diagnosis was challenging or impossible.

The study was conducted in accordance with the Declaration of Helsinki (as revised in 2013). It was approved by the Institutional Review Board of Kyoto Prefectural University of Medicine (ERB-C-1580) and each of the participating hospitals and was registered at the University Medical Hospital Information Network Clinical Trials Registry (UMIN000044048). All patients provided written informed consent before participation in this prospective study. In addition, opt-out informed consent was provided at each hospital where the trial was conducted.

### Definition of Geriatric 8, Charlson Comorbidity Index, and Cancer Cachexia

The cutoff values for the geriatric 8 (G8) and Charlson comorbidity index (CCI) were determined according to previous studies.^14^, ^15^, ^16^ Cancer cachexia was defined as follows: weight loss amounting to more than 5% of the total body weight or a body mass index of less than 20 kg/m^2^ and weight loss amounting to more than 2% of the total body weight within 6 months before treatment initiation, with laboratory results of the following parameters exceeding reference values: C-reactive protein more than 0.5 mg/dL, serum albumin less than 3.2 g/dL, or hemoglobin less than 12 g/dL.^17^

### Safety Analysis

In the safety analysis, AEs were assessed by the physician using the Cancer Institute Common Terminology Criteria for Adverse Events, version 5.0. Severe AEs were defined as febrile neutropenia, grade more than or equal to four hematologic AEs, and grade more than or equal to three nonhematologic AEs.

### Statistical Analysis

Statistical significance was set at *p* value less than 0.05. Fisher’s exact or chi-square test was used to compare categorical variables, as appropriate. We compared continuous variables using the Mann-Whitney *U* test. The Kaplan-Meier method was used to estimate PFS and OS, and differences were compared using the log-rank test. In multivariate analyses, Cox proportional hazards models were used to calculate the hazard ratio (HR) with 95% confidence intervals (CIs). On the basis of previous studies on chemoimmunotherapy, sex, age (≥75 y), ECOG-PS (PS ≥2), and durvalumab regimen were selected as covariates.^7^^,^^8^ EZR statistical software version 1.54 was used for all statistical analyses.^18^

## Results

### Patients’ Characteristics

A total of 46 patients were assessed in the present study between September 2019 and October 2021. Of which, 45 patients were included in the analyses because one patient received chemoradiotherapy. Of the 45 patients, 18 patients were aged more than or equal to 75 years (elderly) and 27 patients were aged less than 75 years (non-elderly) (Table 1). There were no significant differences in background other than age and treatment regimen between the elderly and non-elderly patients. In regard to all patients, the median age was 73 years (range, 50–86 y), 36 patients (80.0%) were male, six patients (13.3%) had a PS of 2, nine (20.0%) patients had cancer cachexia, and 35 patients (77.8%) received the atezolizumab regimen. The median number of maintenance therapy in all patients was two (range: 0–6). The actual initial doses for each patient are found in Supplementary Table 1.

**Table 1:** Patient Characteristic

Characteristics	All Patients (N = 45)	Age ≥ 75 y (n = 18, 40.0%)	Age < 75 y (n = 27, 60.0%)	*p* Value
Age				
Median (range)	73 (50–86)	79.5 (75–86)	69 (50–74)	<0.001
Sex, n (%)				
Male	36 (80.0)	15 (83.3)	21 (77.8)	0.72
Female	9 (20.0)	3 (16.7)	6 (22.2)	
ECOG-performance status, n (%)				
0	10 (22.2)	4 (22.2)	6 (22.2)	0.20^a^
1	29 (64.4)	10 (55.6)	19 (70.4)	
2	6 (13.3)	4 (22.2)	2 (7.4)	
Previous anticancer treatments, n (%)				
Chemoradiotherapy	2 (4.4)	1 (5.6)	1 (3.7)	
Cancer-related surgery	1 (2.2)	0 (0)	1 (3.7)	
Smoking status, n (%)				
Current/former	44 (97.8)	17 (94.4)	27 (100)	0.40
Never	1 (2.2)	1 (5.6)	0 (0)	
BMI				
Median	21.4 (17.5–30.1)	21.4 (18.3–28.8)	21.4 (17.5–30.1)	0.87
Cancer cachexia, n (%)	9 (20.0)	4 (22.2)	5 (18.5)	1.0
Preexisting interstitial lung disease, n (%)	2 (4.4)	1 (5.6)	1 (3.7)	1.0
Charlson comorbidity index				
Median (range)	1 (0–4)	1 (0–4)	1 (0–3)	0.28^b^
G8				
Median (range)	11 (4.5–17)	11 (5–15)	11.5 (4.5–17)	0.99
Regimen, n (%)				
Carboplatin + etoposide + atezolizumab	35 (77.8)	17 (94.4)	18 (66.7)	0.03^c^
Carboplatin + etoposide + durvalumab	8 (17.8)	1 (5.6)	7 (25.9)	
Cisplatin + etoposide + durvalumab	2 (4.4)	0 (0)	2 (7.4)	
Median number of maintenance therapy (range)	2 (0–6)	1 (0–5)	2 (0–6)	0.24

ECOG-PS, Eastern Cooperative Oncology Group performance status; BMI, body mass index; G8, geriatric 8.

a ECOG-PS 2 versus 0 or 1.

b One patient’s data were missing.

c Atezolizumab versus durvalumab.

### Treatment Efficacy

**Figure 1:**
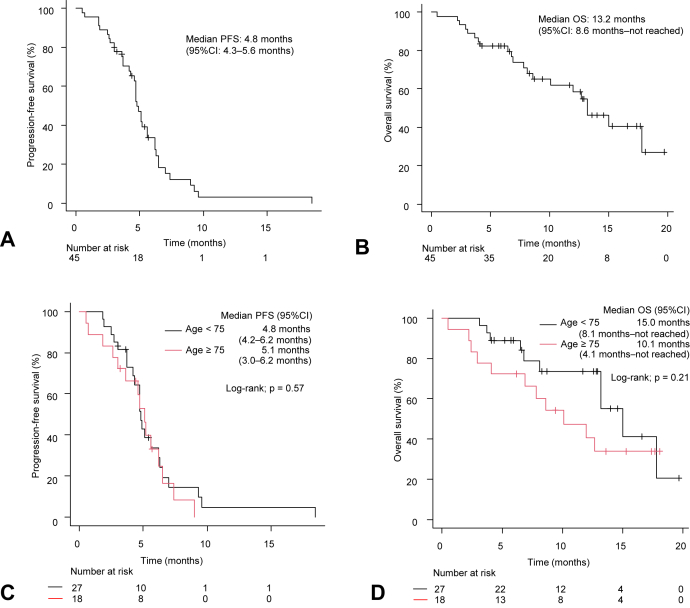
PFS (*A*) and OS (*B*) of PD-L1 inhibitor plus platinum-etoposide chemotherapy in all patients (n = 45). PFS (*C*) and OS (*D*) of PD-L1 inhibitor plus platinum-etoposide chemotherapy compared with age (≥75 y versus <75 y). CI, confidence interval; HR, hazard ratio; OS, overall survival; PD-L1, programmed death-ligand 1; PFS, progression-free survival.

The median follow-up time was 12.2 months (range: 4.1–19.7 mo) for censored cases. The median PFS and OS were 4.8 months (95% CI: 4.3–5.6 mo) and 13.2 months (95% CI: 8.6 mo–not reached), respectively (Fig. 1*A* and *B*).

There was no significant difference in PFS and OS between the elderly and non-elderly patients who received PD-L1 inhibitor plus platinum-etoposide chemotherapy (PFS: 5.1 mo [95% CI: 3.0–6.2 mo] versus 4.8 mo [95% CI: 4.2–6.2 mo], log-rank test *p* = 0.57; OS: 10.1 mo [95% CI: 4.1 mo–not reached] versus 15.0 mo [95% CI: 8.1 mo–not reached], log-rank test *p* = 0.21, respectively) (Fig. 1*C* and *D*). Rates of PFS and OS at age cutoffs of 70 and 80 years were found in Supplementary Figure 2.

**Figure 2:**
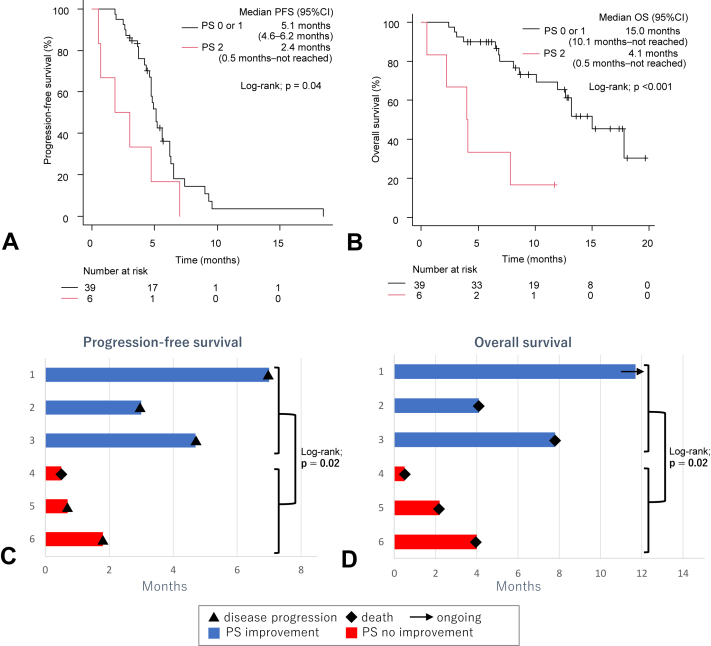
(*A*) Kaplan-Meier curve for PFS of patients with ES-SCLC compared with PS (0 or 1 versus 2). (*B*) Kaplan-Meier curve for OS of patients with ES-SCLC compared with performance status (0 or 1 versus 2). (*C*) PFS of PD-L1 inhibitor plus platinum-etoposide chemotherapy in ES-SCLC patients with a PS of 2. (*D*) OS of PD-L1 inhibitor plus platinum-etoposide chemotherapy in ES-SCLC patients with a PS of 2. CI, confidence interval; ES-SCLC, extensive- stage SCLC; HR, hazard ratio; OS, overall survival; PD-L1, programmed death-ligand- 1, PFS, progression-free survival; PS, performance status.

Patients with a PS of 2, however, had significantly shorter PFS and OS after treatment with PD-L1 inhibitor plus platinum-etoposide chemotherapy, as compared with patients with a PS or 0 or 1 (PFS: 2.4 mo [95% CI: 0.5 mo–not reached] versus 5.1 mo [95% CI: 4.6–6.2 mo], log-rank test *p* = 0.04; OS: 4.1 mo [95% CI: 0.5 mo–not reached] versus 15.0 mo [95% CI: 4.1 mo–not reached], log-rank test *p* < 0.001, respectively) (Fig. 2*A*, *B*). Multivariate analysis identified a PS of 2 as a significant independent prognostic factor of PFS (HR: 3.83, 95% CI: 1.40–10.4, *p* = 0.008) and OS (HR = 7.31, 95% CI: 2.19–24.4, *p* = 0.001) (Table 2). Of the six patients with a PS of 2, three (50.0%) had improvement in PS during chemoimmunotherapy (Supplementary Fig. 3*C*). Patients with a PS of 2 were further divided into the following two groups: those who had improvement in PS after the initiation of PD-L1 inhibitor plus platinum-etoposide chemotherapy and those who did not. There were significant differences in PFS and OS after treatment with PD-L1 inhibitor plus platinum-etoposide chemotherapy among the two groups (log-rank test *p* = 0.02 and *p* = 0.02, respectively) (Fig. 2*C* and *D*).

**Table 2:** Cox Proportional Hazard Models for Progression-Free Survival and Overall Survival in Patients With Extensive-Stage SCLC Who Received PD-L1 Inhibitor Plus Platinum-Etoposide Chemotherapy in Multivariate Analysis

Items	Multivariate Analysis			
	Progression-Free Survival		Overall Survival	
	HR (95% CI)	*p* Value	HR (95% CI)	*p* Value
Age ≥75 y (vs. age < 75 y)	1.08 (0.53–2.23)	0.83	1.13 (0.45–2.82)	0.80
Male sex (vs. female sex)	0.47 (0.19–1.17)	0.10	0.98 (0.27–3.51)	0.97
ECOG-PS 2 (vs. ECOG-PS 0 or 1)	3.83 (1.40–10.4)	0.008	7.31 (2.19–24.4)	0.001
Durvalumab regimen (vs. atezolizumab regimen)	0.46 (0.18–1.18)	0.11	0.17 (0.02–1.42)	0.10

CI, confidence interval; ECOG-PS, Eastern Cooperative Oncology Group performance status; HR, hazard ratio; PD-L1, programmed death-ligand 1.

The group of patients with a PS of 2 that did not have improvement in PS after the initiation of PD-L1 inhibitor plus platinum-etoposide chemotherapy had significantly shorter PFS than the group of patients with a PS of 0 or 1 (0.7 mo [95% CI: 0.5 mo–not reached] versus 5.1 mo [95% CI: 4.6–6.2 mo], log-rank test *p* < 0.001) (Supplementary Fig. 3*D*). Nevertheless, the group of patients with a PS of 2 that had improvement in PS after the initiation of PD-L1 inhibitor plus platinum-etoposide chemotherapy did not have any significant differences in PFS as compared with the patients with a PS of 0 or 1 (4.7 mo [95% CI: 3.0 mo–not reached] versus 5.1 mo [95% CI: 4.6–6.2 mo], log-rank test *p* = 0.69). All patients with a PS of 2 who had improvement in PS achieved an objective response, whereas all patients who did not have improvement in PS did not achieve an objective response (data not found). In contrast, G8 less than or equal to 14 and CCI more than or equal to 2 did not significantly influence PFS (4.7 mo [95% CI: 3.7–5.6 mo] versus 5.6 mo [95% CI: 5.2 mo–not reached], log-rank test *p* = 0.26 and 6.2 mo [95% CI: 3.0–6.5 mo] versus 4.7 mo [95% CI: 4.2–5.2 mo], log-rank test *p* = 0.37) and OS (13.2 mo [95% CI: 8.1–17.8 mo] versus not reached [95% CI: not reached], log-rank test *p* = 0.22 and 12.7 mo [95% CI: 4.1 mo–not reached] versus 13.2 mo [95% CI: 6.9 mo–not reached], log-rank test *p* = 0.87) in patients who received PD-L1 inhibitor plus platinum-etoposide chemotherapy (Supplementary Fig. 4*A*–*D*). Patients with cancer cachexia tended to have a shorter OS than those without cancer cachexia, and the difference was significant when the analysis was limited to patients with a PS 0 or 1 (12.0 mo [95% CI: 2.2 mo–not reached] versus 15.0 mo [95% CI: 8.6 mo–not reached], log-rank test *p* = 0.07 and 12.0 mo [95% CI: 2.4 mo–not reached] versus 17.8 mo [95% CI: 12.7 mo–not reached], log-rank test *p* = 0.02, respectively) (Supplementary Figs. 4F and 5*F*).

### Treatment Safety

Overall, 27 patients (60.0%) experienced severe AEs (Supplementary Table 2). There were no significant differences in patient characteristics between those who developed serious AEs and those who did not. Six patients (13.3%) discontinued all types of treatment owing to AEs (Table 3). Of which, three patients discontinued treatment during the induction phase and three patients discontinued treatment during the maintenance phase. The most frequent AEs leading to discontinuation of all kinds of treatment were pneumonitis (n = 3, 50.0%). Treatment discontinuation related to AEs occurred at a significantly higher rate in elderly patients than in non-elderly patients (median [range] 81.5 [72–84] versus 72 [50–86], respectively, *p* = 0.009). In the elderly group, two patients (11.1%) died of treatment-related AEs (Table 4). The elderly group had a significantly higher rate of discontinuation of all kinds of treatment owing to AEs, as compared with the non-elderly group (*p* = 0.03). Table 5 lists the severity of AEs, such as pneumonitis, between different age groups (≥75 y versus <75 y). Overall, four patients (8.9%) experienced pneumonitis. The elderly group had a higher rate of pneumonitis than the non-elderly group (16.7% versus 3.7%). Treatment-related severe AEs and pneumonitis at age cutoff of 70 are found in Supplementary Table 3.

**Table 3:** Comparison Between Patients With and Without Discontinuation Due to AEs

Characteristics	With Discontinuation Due to AEs (n = 6, 13.3%)	Without Discontinuation Due to AEs (n = 39, 86.7%)	*p* Value
Age			
Median (range)	81.5 (72–84)	72 (50–86)	0.009
Sex, n (%)			
Male	5 (83.3)	31 (79.5)	1.0
Female	1 (16.7)	8 (20.5)	
ECOG-PS, n (%)			
0	1 (16.7)	9 (23.1)	1.0^a^
1	4 (66.7)	25 (64.1)	
2	1 (16.7)	5 (12.8)	
Cancer cachexia, n (%)	0 (0)	9 (23.1)	0.32
Smoking status, n (%)			
Current/former	5 (83.3)	39 (100)	0.13
Never	1 (16.7)	0 (0)	
BMI			
Median	24.2 (20.8–28.1)	21.0 (17.5–30.1)	0.20
Charlson comorbidity index			
Median (range)	2 (0–3)	1 (0–4)	0.14
G8			
Median (range)	11.5 (11–14)	11 (4.5–17)	0.46
Regimen, n (%)			
Carboplatin + etoposide + atezolizumab	6 (100)	29 (74.4)	0.31^b^
Carboplatin + etoposide + durvalumab	0 (0)	8 (20.5)	
Cisplatin + etoposide + durvalumab	0 (0)	2 (5.1)	

AE, adverse event; ECOG-PS, Eastern Cooperative Oncology Group performance status; BMI, body mass index; G8, geriatric 8.

a ECOG-PS 2 versus 0 or 1.

b Atezolizumab versus durvalumab.

**Table 4:** Treatment-Related Severe Adverse Events Compared With Age (≥75 y Versus <75 y)

AEs	≥75 y (n = 18, 40.0%)	<75 y (n = 27, 60.0%)	*p* Value
Nonhematological AEs, grade ≥4, n (%)			
Febrile neutropenia	3 (16.7)	1 (3.7)	0.29
Neutropenia	8 (44.4)	17 (63.0)	0.24
Hematological AEs, grade ≥3, n (%)			
Fatigue	1 (5.6)	0 (0)	0.40
Anorexia	1 (5.6)	1 (3.7)	1.0
Urinary tract infection	0 (0)	1 (3.7)	1.0
Pneumonitis	1 (5.6)	0 (0)	0.40
Myasthenia gravis	0 (0)	1 (3.7)	1.0
Discontinuation of all treatment due to AEs	5 (27.8)	1 (3.7)	0.03
Event leading to death	2^a^ (11.1)	0 (0)	0.16

AE, adverse event.

a Sepsis in two patients.

**Table 5:** Severity of AEs of Pneumonitis Compared With Age (≥75 y Versus <75 y)

Pneumonitis	Grade 1	Grade 2	Grade 3	Grade 4	Grade 5
Total, n (%)					
(n = 45)	1 (2.2)	2 (4.4)	1 (2.2)	0 (0)	0 (0)
≥75 y (n = 18)	1 (5.6)	1 (5.6)	1 (5.6)	0 (0)	0 (0)
<75 y (n = 27)	0 (0)	1 (3.7)	0 (0)	0 (0)	0 (0)

### Treatment After Disease Progression

At the data cutoff date, 38 patients developed disease progression. Two of them had died owing to treatment-related causes, and two had not yet decided on their subsequent therapy, shortly after disease progression. Of the 34 patients, 27 (79.4%) received the first subsequent therapy. The group that did not receive subsequent therapy tended to be older than the group that did receive first subsequent therapy (*p* = 0.07) (Supplementary Table 4A). Supplementary Table 4B lists the treatment administered after disease progression in 13 patients of the elderly groups and 21 patients of the non-elderly group. Best supportive care without the first subsequent therapy was provided for four patients (30.8%) in the elderly group and three patients (13.8%) in the non-elderly group. Four patients (19.0%) in the non-elderly group received platinum doublet therapy whereas none of the patients in the elderly group underwent this treatment.

## Discussion

To the best of our knowledge, this is the first prospective real-world study that investigated the efficacy and safety of PD-L1 inhibitor plus platinum-etoposide chemotherapy in patients with ES-SCLC, stratified on the basis of age and ECOG-PS.

Aging is a process in which the accumulation of genetic and environmental factors leads to a decline in the function of various organs, along with the regenerative capacity of tissues.^19^ In the IMpower133 trial of carboplatin-etoposide plus atezolizumab for the treatment of ES-SCLC, the safety and efficacy of the treatment were favorable for elderly patients.^20^ Our current results reveal that this treatment is effective for patients with ES-SCLC, regardless of age, in the real-world setting. Nevertheless, in regard to safety, the rate of treatment discontinuation owing to AEs was higher with increasing age. We particularly found that the incidence of pneumonitis was higher in elderly patients with ES-SCLC than in non-elderly patients; therefore, careful attention should be paid when administering this treatment to this population. Furthermore, an increase in age led to a decrease in the rate of patients receiving the first subsequent treatment. Imai et al.^21^ reported that the first subsequent treatment may affect OS in elderly patients with ES-SCLC. Although the difference was insignificant, elderly patients with ES-SCLC had a shorter OS than non-elderly patients. As per a previous report, elderly patients with SCLC are reported to less likely receive chemotherapy than non-elderly patients.^22^ In addition, discontinuation of PD-L1 inhibitor plus platinum-etoposide chemotherapy owing to AEs may have contributed to the poor OS by worsening the patient's general condition and making it more difficult to receive the first subsequent treatment.

In patients with nonsquamous NSCLC, poor ECOG-PS has been found to be a poor prognostic factor in patients receiving pembrolizumab plus chemotherapy.^10^ In a retrospective data analysis, chemotherapy plus atezolizumab for patients with ES-SCLC with a PS of more than or equal to 2 was reported to be feasible; however, the OS was short, which is consistent with our present observations.^23^

In this study, PFS and OS after platinum plus etoposide chemotherapy in patients with ES-SCLC with a PS of 2 were significantly poorer than those in patients with ES-SCLC with a PS of 0 or 1. The subset of patients with a PS of 2 at baseline had a relatively good therapeutic effect when they transitioned to a PS of 0 or 1 during treatment. Previous reports revealed that an improvement in PS was a good indicator of topotecan therapy in patients with recurrent SCLC and a poor PS.^24^ Improvement of PS may be strongly associated with the therapeutic effect of PD-L1 inhibitor plus platinum-etoposide chemotherapy. It has been suggested that cancer cachexia may be associated with poor clinical outcomes after administering chemoimmunotherapy in patients with NSCLC.^17^ In this study, patients with ES-SCLC with cancer cachexia tended to have shorter OS than those without cancer cachexia. Furthermore, cancer cachexia was a poor prognostic factor for OS when evaluated only in patients with ES-SCLC with a PS of 0 or 1. Interventions for cancer cachexia in patients with ES-SCLC may be promising for the improvement of clinical outcomes.

Our study had some limitations. First, the sample size was small, including fewer patients with a PS of 2. Second, this study was conducted only among Japanese patients, limiting its generalizability. Finally, the follow-up period was also short to evaluate OS.

In conclusion, treatment of platinum plus etoposide chemotherapy for elderly patients with ES-SCLC is effective, but it requires caution regarding AEs. Although the efficacy of platinum plus etoposide chemotherapy in patients with ES-SCLC with a PS of 2 is unfavorable, it may be considered in patients for whom PS improvement can be expected with treatment. Further large-scale clinical investigations are required to confirm these observations.

## CRediT Authorship Contribution Statement

**Kenji Morimoto and Tadaaki Yamada:** Study conception and design.

**Kenji Morimoto, Takayuki Takeda, Shinsuke Shiotsu, Koji Date, Taishi Harada, Nobuyo Tamiya, Yusuke Chihara, Osamu Hiranuma, Takahiro Yamada, Hibiki Kanda, and Takayuki Nakano:** Obtained the clinical data.

**Kenji Morimoto, Tadaaki Yamada, Yoshie Morimoto, Masahiro Iwasaku, Shinsaku Tokuda, and Koichi Takayama**: Data Interpretation.

**Kenji Morimoto, Tadaaki Yamada, and Koichi Takayama**: Manuscript preparation.

The final version of the manuscript was read and approved by all the authors.

## Ethics Approval

The authors conducted the study in accordance with the Declaration of Helsinki (as revised in 2013). This study was approved by the institutional review board in Kyoto Prefectural University of Medicine (ERB-C-1580) and those of each respective hospital and registered at the University Medical Hospital Information Network Clinical Trials Registry (UMIN000044048). Opt-out informed consent was provided at each hospital that the trial was conducted. Written informed consent was obtained from all participants.

## Availability of Data and Material

The data sets generated during the current study are not publicly available owing to ethical constraints but are available from the corresponding author on reasonable request.
