# Supplementary material for: Efficacy and Safety of Programmed Death-Ligand 1 Inhibitor Plus Platinum-Etoposide Chemotherapy in Patients With Extensive-Stage SCLC: A Prospective Observational Study
Source: JTO Clin Res Rep. 2022 Jun 8;3(7):100353. doi: 10.1016/j.jtocrr.2022.100353 (PMC9250020; doi:10.1016/j.jtocrr.2022.100353)
Supplement: Supplementary Figures [file mmc1.pdf]

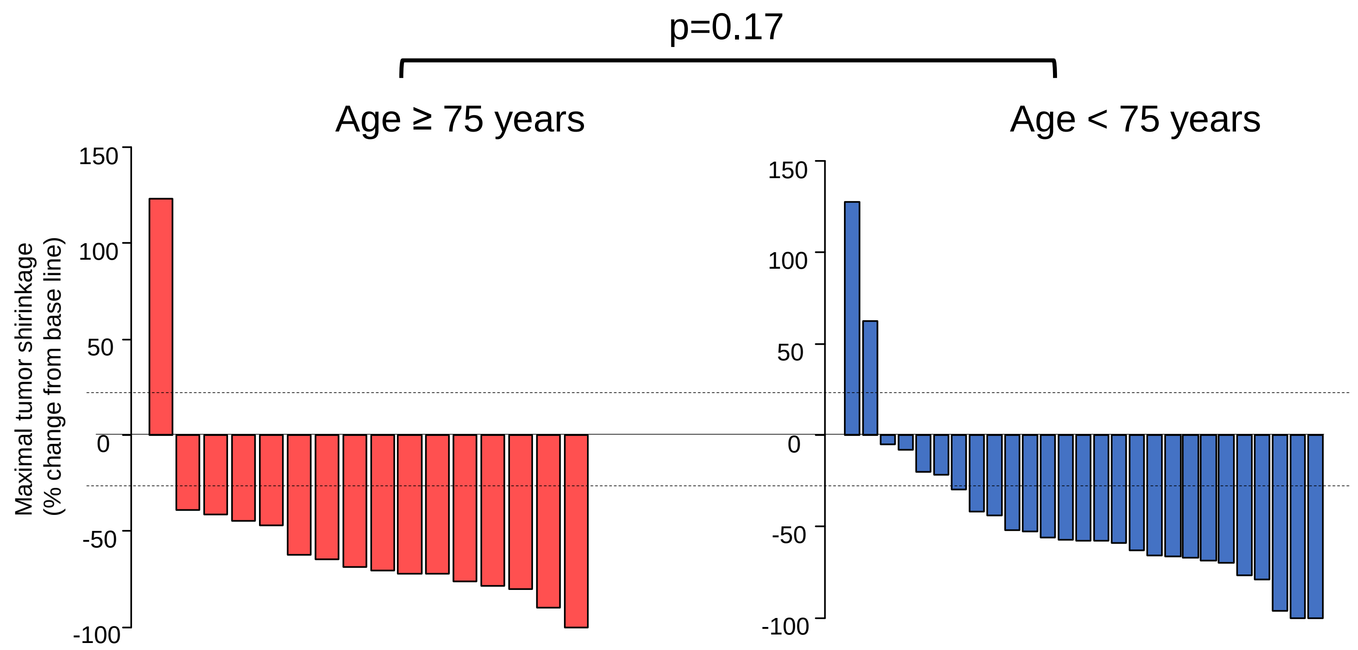

	CR	PR	SD	PD	NE
All patients	6.7%	75.6%	8.9%	6.7%	2.2%
Age < 75 years	7.4%	70.4%	14.8%	7.4%	0.0%
Age ≥ 75 years	5.6%	83.3%	0.0%	5.6%	5.6%

p = 0.44

Comparison of ORR

Supplementary Figure 1.

Waterfall plot of patients with ES-SCLC treated with PD-L1 inhibitor plus platinum-etoposide chemotherapy .

The maximum tumor shrinkage rate relative to baseline in 43 patients with ES-SCLC treated with PD-L1 inhibitor plus platinum-etoposide chemotherapy. Two patients had missing data.

Red bars indicate the maximum tumor shrinkage for patients aged ≥75 (N=16); blue bars for patients aged <75 years (n=27).

There was no significant difference in the median tumor shrinkage rate (69.9% and 58.0%, respectively, p=0.17).

PD-L1, programmed death-ligand 1; PFS, progression-free survival; ES-SCLC, extensive stage-small cell lung cancer; ORR, overall response rate; CR, complete response; PR, partial response; SD, stable disease; PD, progressive disease; NE, not evaluable.

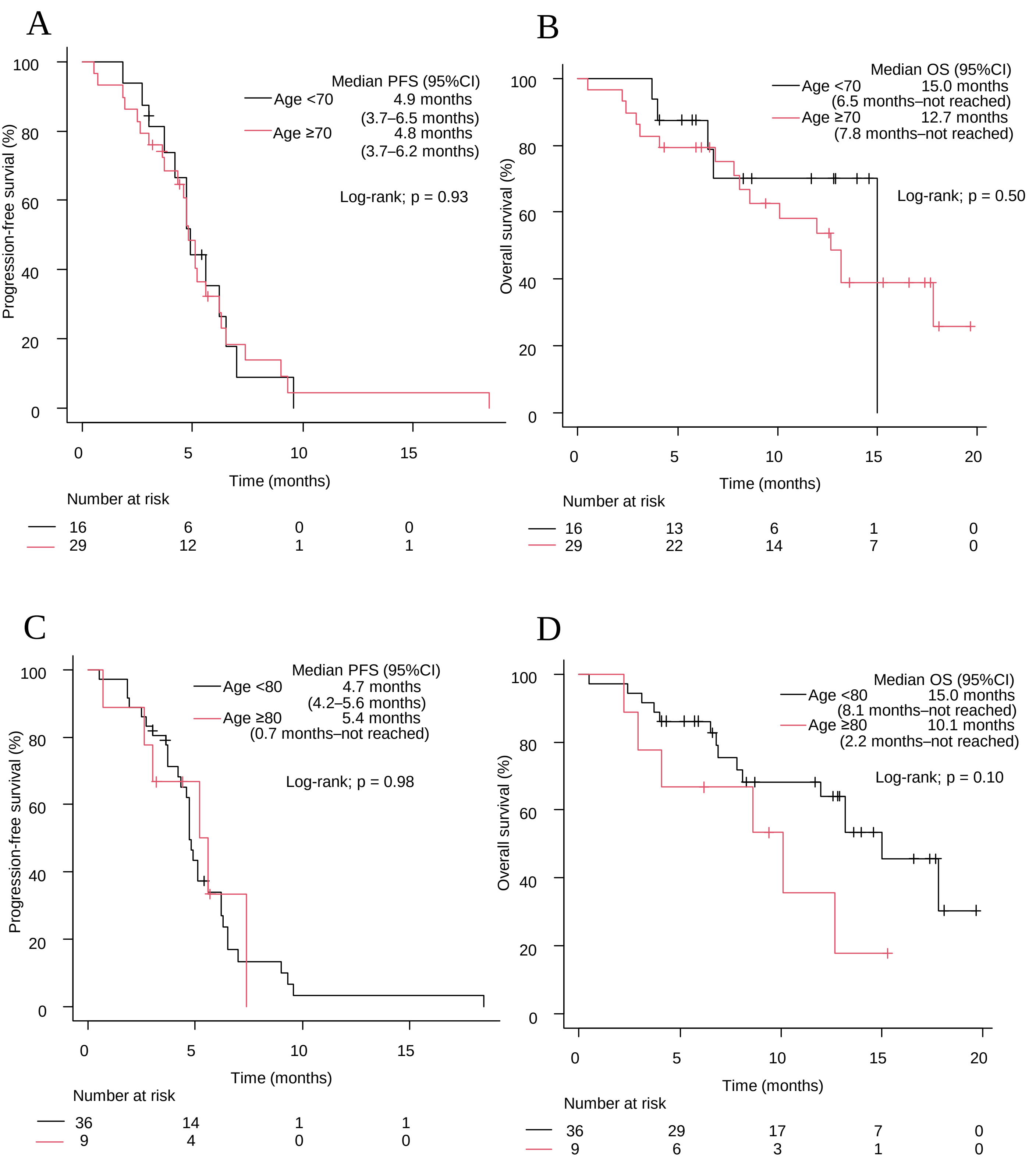

Supplementary Figure 2.

PFS (A) and OS (B) of PD-L1 inhibitor plus platinum-etoposide chemotherapy compared with age (≥70 years versus <70 years).

PFS (C) and OS (D) of PD-L1 inhibitor plus platinum-etoposide chemotherapy compared with age (≥80 years versus <80 years).PD-L1, programmed death-ligand 1; PFS, progression-free survival; OS, overall survival; HR, hazard ratio; CI, confidence interval.

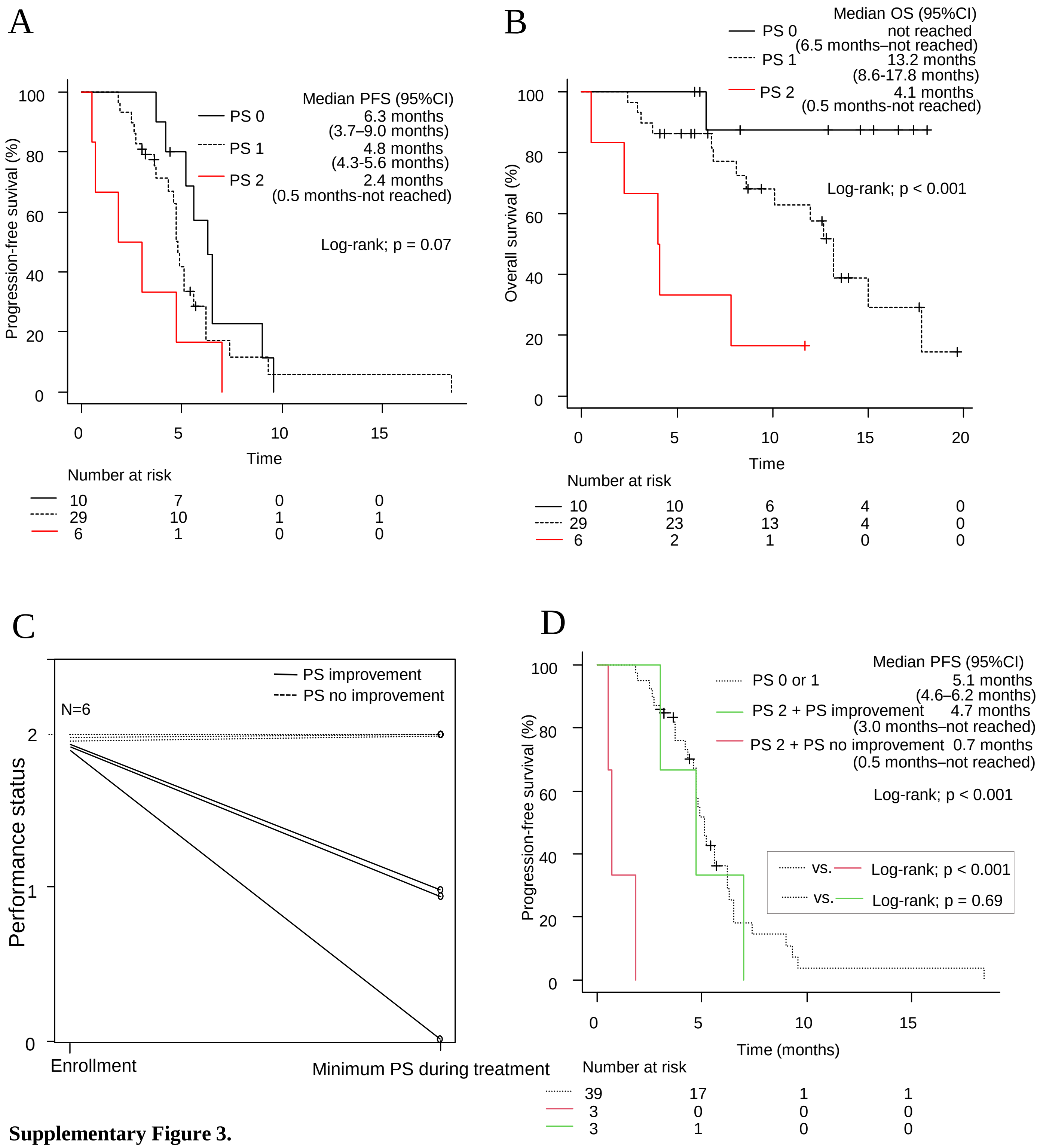

Supplementary Figure 3.

(A) Kaplan-Meier curves for PFS of patients with ES-SCLC stratified by PS.

(B) Kaplan-Meier curves for OS of patients with ES-SCLC stratified by PS.

(C) Change in the PS of each patient during PD-L1 inhibitor plus platinum-etoposide chemotherapy.

Each line shows the change in PS of a patient from baseline to their best status during PD-L1 inhibitor plus platinum-etoposide chemotherapy.

(D) Kaplan-Meier curve for PFS of patients with ES-SCLC compared according to the PS (0 or 1 vs. 2), and improvement of PS.

ES-SCLC, extensive stage small cell lung cancer; PFS, progression-free survival; PD-L1, programmed death-ligand 1; PS, performance status; OS, overall survival; HR, hazard ratio; CI, confidence interval; vs, versus.

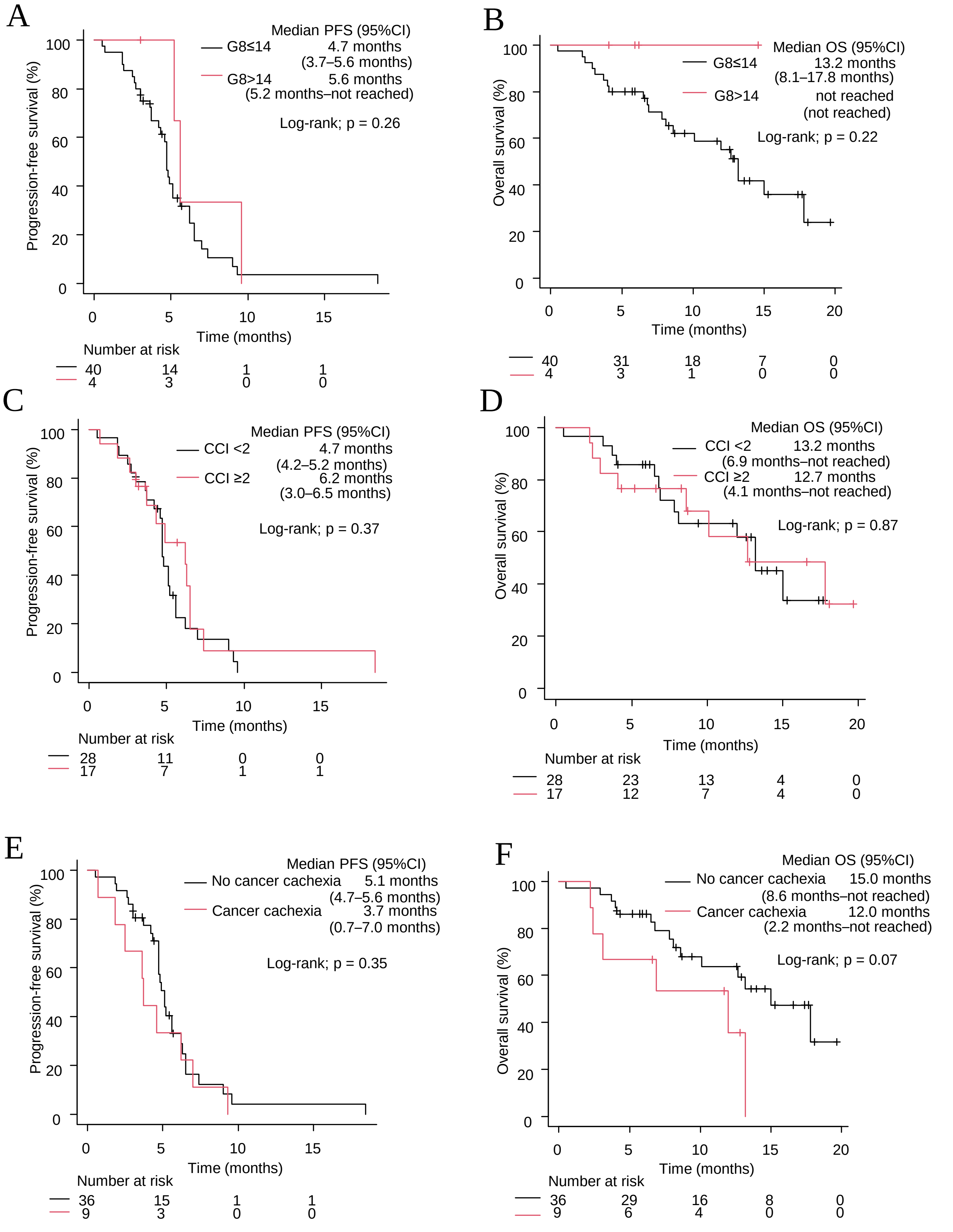

Supplementary Figure 4.

PFS (A) and OS (B) of PD-L1 inhibitor plus platinum-etoposide chemotherapy according to CCI status.
PFS (C) and OS (D) of PD-L1 inhibitor plus platinum-etoposide chemotherapy according to G8 status
PFS (E) and OS (F) of PD-L1 inhibitor plus platinum-etoposide chemotherapy according to the presence of cancer cachexia.
PD-L1, programmed death-ligand 1; PFS, progression-free survival; OS, overall survival; HR, hazard ratio; CI, confidence interval; CCI, Charlson comorbidity index; G8, geriatric 8.

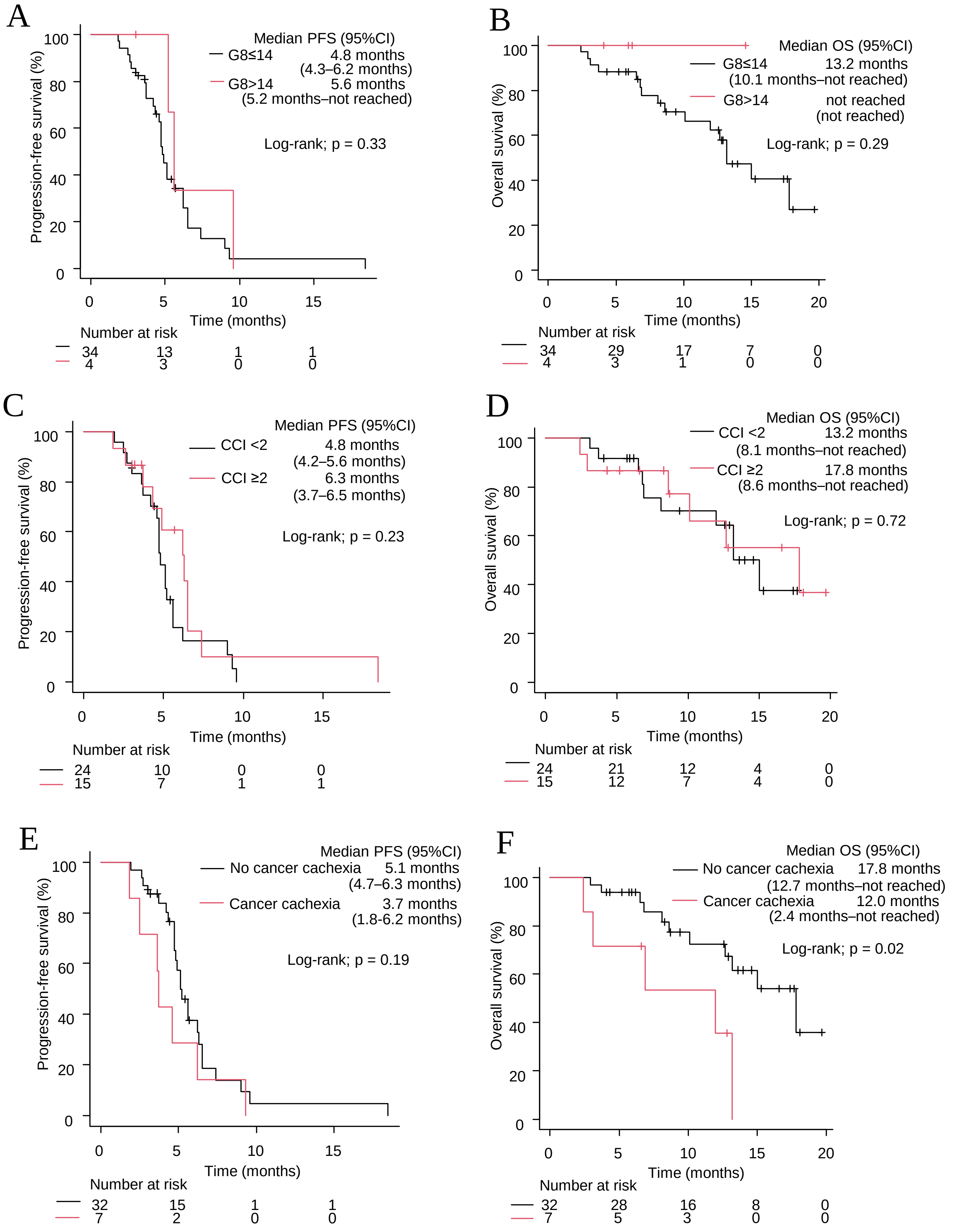

Supplementary Figure 5.
PFS (A) and OS (B) of PD-L1 inhibitor plus platinum-etoposide chemotherapy according to CCI status excluding patients with a PS of 2. PFS (C) and OS (D) of PD-L1 inhibitor plus platinum-etoposide chemotherapy according to G8 status excluding patients with a PS of 2. PFS (E) and OS (F) of PD-L1 inhibitor plus platinum-etoposide chemotherapy according to the presence of cancer cachexia excluding patients with a PS of 2.
PD-L1, programmed death-ligand 1; PFS, progression-free survival; OS, overall survival; HR, hazard ratio; CI, confidence interval; CCI, Charlson comorbidity index; G8, geriatric 8; PS, performance status.
