# Supplementary material for: Efficacy and Safety of Programmed Death-Ligand 1 Inhibitor Plus Platinum-Etoposide Chemotherapy in Patients With Extensive-Stage SCLC: A Prospective Observational Study
Source: JTO Clin Res Rep. 2022 Jun 8;3(7):100353. doi: 10.1016/j.jtocrr.2022.100353 (PMC9250020; doi:10.1016/j.jtocrr.2022.100353)
Supplement: Supplementary Tables [file mmc2.docx]

**Supplementary Table 1. Actual initial doses for each patient**

| No. | Age (y) | Sex | ECOG-PS | Carboplatin  (AUC) | Cisplatin  (mg/㎡) | Etoposide  (mg/㎡) | Atezolizumab  (mg) | Durvalumab  (mg) |
| --- | --- | --- | --- | --- | --- | --- | --- | --- |
| 1 | 60 | M | 0 | 5 |  | 100 | 1200 |  |
| 2 | 70 | F | 1 | 5 |  | 80 | 1200 |  |
| 3 | 82 | M | 1 | 5 |  | 100 | 1200 |  |
| 4 | 72 | M | 1 | 5 |  | 80 | 1200 |  |
| 5 | 71 | M | 1 | 5 |  | 100 | 1200 |  |
| 6 | 75 | M | 0 | 5 |  | 83 | 1200 |  |
| 7 | 71 | F | 1 | 5 |  | 100 | 1200 |  |
| 8 | 66 | M | 1 | 5 |  | 100 | 1200 |  |
| 9 | 75 | M | 1 | 5 |  | 80 | 1200 |  |
| 10 | 79 | M | 0 | 4 |  | 70 | 1200 |  |
| 11 | 83 | M | 1 | 5 |  | 80 | 1200 |  |
| 12 | 74 | M | 1 | 5 |  | 80 | 1200 |  |
| 13 | 76 | F | 1 | 5 |  | 100 | 1200 |  |
| 14 | 73 | F | 0 | 5 |  | 80 | 1200 |  |
| 15 | 76 | M | 1 | 5 |  | 100 | 1200 |  |
| 16 | 69 | M | 1 | 5 |  | 80 | 1200 |  |
| 17 | 86 | M | 2 | 5 |  | 56 | 1200 |  |
| 18 | 84 | F | 0 | 5 |  | 63 | 1200 |  |
| 19 | 82 | M | 1 | 4 |  | 80 | 1200 |  |
| 20 | 51 | M | 0 |  | 75 | 80 |  | 1500 |
| 21 | 77 | M | 2 | 5 |  | 100 | 1200 |  |
| 22 | 83 | M | 0 | 5 |  | 80 | 1200 |  |
| 23 | 69 | M | 1 | 4 |  | 80 |  | 1500 |
| 24 | 75 | M | 1 | 5 |  | 75 | 1200 |  |
| 25 | 67 | M | 0 | 5 |  | 80 | 1200 |  |
| 26 | 69 | M | 1 | 5 |  | 80 | 1200 |  |
| 27 | 73 | M | 1 | 5 |  | 80 | 1200 |  |
| 28 | 72 | M | 1 | 5 |  | 80 | 1200 |  |
| 29 | 59 | M | 2 | 5 |  | 100 |  | 1500 |
| 30 | 69 | M | 2 | 5 |  | 80 | 1200 |  |
| 31 | 76 | M | 2 | 5 |  | 80 |  |  |
| 32 | 83 | F | 1 | 5 |  | 72 |  | 1500 |
| 33 | 81 | M | 1 | 4 |  | 80 | 1200 |  |
| 34 | 66 | M | 1 | 5 |  | 100 | 1200 |  |
| 35 | 69 | F | 0 | 5 |  | 100 |  | 1500 |
| 36 | 79 | M | 1 | 5 |  | 80 | 1200 |  |
| 37 | 59 | M | 1 | 5 |  | 100 |  | 1500 |
| 38 | 74 | F | 1 | 5 |  | 85 |  | 1500 |
| 39 | 73 | M | 1 | 5 |  | 100 | 1200 |  |
| 40 | 69 | F | 0 | 5 |  | 80 | 1200 |  |
| 41 | 69 | M | 1 | 5 |  | 100 | 1200 |  |
| 42 | 67 | M | 1 | 5 |  | 100 |  | 1500 |
| 43 | 72 | M | 1 | 5 |  | 80 | 1200 |  |
| 44 | 50 | M | 1 |  | 80 | 100 |  | 1500 |
| 45 | 80 | M | 2 | 4 |  | 80 | 1200 |  |

AUC, area under the concentration-tie curve; ECOG-PS, Eastern Cooperative Oncology Group performance status.
