# Supplementary material for: Efficacy and Safety of Programmed Death-Ligand 1 Inhibitor Plus Platinum-Etoposide Chemotherapy in Patients With Extensive-Stage SCLC: A Prospective Observational Study
Source: JTO Clin Res Rep. 2022 Jun 8;3(7):100353. doi: 10.1016/j.jtocrr.2022.100353 (PMC9250020; doi:10.1016/j.jtocrr.2022.100353)
Supplement: Supplementary Tables [file mmc3.docx]

**Supplementary Table 2**

Comparison between patients with and without severe AEs.

| Characteristics | With severe AEs  (n = 27, 60.0%) | Without severe AEs  (n = 18, 40.0%) | p-value |
| --- | --- | --- | --- |
| Age |  |  |  |
| Median (range) | 74 (51-86) | 71 (50-83) | 0.11 |
| Sex |  |  |  |
| Male | 21 (77.8%) | 15 (83.3%) | 0.72 |
| Female | 6 (22.2%) | 3 (16.7%) |  |
| ECOG-PS |  |  |  |
| 0 | 4 (14.8%) | 6 (33.3%) | 0.67^a^ |
| 1 | 20 (74.1%) | 9 (50.0%) |  |
| 2 | 3 (11.1%) | 3 (16.7%) |  |
| Cancer cachexia | 4 (14.8%) | 5 (27.8%) | 0.45 |
| Smoking status |  |  |  |
| Current/Former | 26 (96.3%) | 18 (100%) | 1.0 |
| Never | 1 (3.7%) | 0 (0%) |  |
| BMI |  |  |  |
| Median | 21.4 (18.1-30.1) | 21.2 (17.5-28.8) | 1.0 |
| Charlson comorbidity index |  |  |  |
| Median (range) | 1 (0-4) | 1 (0-3) | 0.99 |
| G8 |  |  |  |
| Median (range) | 11.5 (4.5-15.5) | 11 (5-17) | 0.78 |
| Regimen |  |  |  |
| Carboplatin + etoposide + atezolizumab | 19 (70.4%) | 16 (88.9%) | 0.27^b^ |
| Carboplatin + etoposide + durvalumab | 7 (25.9%) | 1 (5.6%) |  |
| Cisplatin + etoposide + durvalumab | 1 (3.7%) | 1 (5.6%) |  |

^a^ ECOG-PS 2 versus 0 or 1. ^b^ atezolizumab versus durvalumab. AE, adverse event; ECOG-PS, Eastern Cooperative Oncology Group performance status; BMI, body mass index; G8, geriatric 8
