# Supplementary material for: Efficacy and Safety of Programmed Death-Ligand 1 Inhibitor Plus Platinum-Etoposide Chemotherapy in Patients With Extensive-Stage SCLC: A Prospective Observational Study
Source: JTO Clin Res Rep. 2022 Jun 8;3(7):100353. doi: 10.1016/j.jtocrr.2022.100353 (PMC9250020; doi:10.1016/j.jtocrr.2022.100353)
Supplement: Supplementary Tables [file mmc4.docx]

**Supplementary Table 3**

A. Treatment related severe adverse events compared with age (≥70 years versus <70 years).

|  | ≥70 years  (n = 29, 64.4%) | <70 years  (n = 16, 35.6%) | p-value |
| --- | --- | --- | --- |
| Non-hematological AEs, grade ≥4 |  |  |  |
| Febrile neutropenia | 3 (10.3%) | 1 (6.3%) | 1.0 |
| Neutropenia | 13 (44.8%) | 12 (75.0%) | 0.07 |
| Hematological AEs, grade ≥3 |  |  |  |
| Fatigue | 1 (3.4%) | 0 (0%) | 1.0 |
| Anorexia | 1 (3.4%) | 1 (6.3%) | 1.0 |
| Urinary tract infection | 1 (3.4%) | 0 (0%) | 1.0 |
| Pneumonitis | 1 (3.4%) | 0 (0%) | 1.0 |
| Myasthenia gravis | 1 (3.4%) | 0 (0%) | 1.0 |
| Discontinuation of all treatment  due to AEs | 6 (27.8%) | 0 (0%) | 0.07 |
| Event leading to death | 2^a^ (6.9%) | 0 (0%) | 0.53 |

^a^ Sepsis in two patients. AE, adverse event.

B. Severity of AEs of pneumonitis compared with age (≥70 years versus <70 years).

| Pneumonitis | Grade 1 | Grade 2 | Grade 3 | Grade 4 | Grade 5 |
| --- | --- | --- | --- | --- | --- |
| ≥70 years  (n = 29) | 1 (3.4%) | 1 (3.4%) | 1 (3.4%) | 0 (0%) | 0 (0%) |
| <70 years  (n = 16) | 0 (0%) | 1 (6.3%) | 0 (0%) | 0 (0%) | 0 (0%) |
