# Supplementary material for: Efficacy and Safety of Programmed Death-Ligand 1 Inhibitor Plus Platinum-Etoposide Chemotherapy in Patients With Extensive-Stage SCLC: A Prospective Observational Study
Source: JTO Clin Res Rep. 2022 Jun 8;3(7):100353. doi: 10.1016/j.jtocrr.2022.100353 (PMC9250020; doi:10.1016/j.jtocrr.2022.100353)
Supplement: Supplementary Tables [file mmc5.docx]

**Supplementary Table 4**

A. Comparison between patients with and without first subsequent therapy

| Characteristics | With first subsequent therapy  (n = 27, 79.4%) | Without first subsequent therapy  (n = 7, 20.6%) | p-value |
| --- | --- | --- | --- |
| Age |  |  |  |
| Median (range) | 71 (51-86) | 76 (69-83) | 0.07 |
| Sex |  |  |  |
| Male | 21 (77.8%) | 6 (85.7%) | 1.0 |
| Female | 6 (22.2%) | 1 (14.3%) |  |
| ECOG-performance status |  |  |  |
| 0 | 7 (25.9%) | 1 (14.3%) | 1.0^a^ |
| 1 | 16 (59.3%) | 5 (71.4%) |  |
| 2 | 4 (14.8%) | 1 (14.3%) |  |
| Cancer cachexia | 6 (22.2%) | 3 (42.9%) | 0.35 |
| Smoking status |  |  |  |
| Current/Former | 27 (100%) | 7 (100%) | 1.0 |
| Never | 0 (0%) | 0 (0%) |  |
| BMI |  |  |  |
| Median | 20.9 (17.5-28.2) | 20.9 (17.5-28.2) | 0.88 |
| Charlson comorbidity index |  |  |  |
| Median (range) | 1 (0-3) | 2 (0-4) | 0.14 |
| G8 |  |  |  |
| Median (range) | 11 (8-17) | 10 (5-15) | 0.25 |
| Regimen |  |  |  |
| Carboplatin + etoposide + atezolizumab | 20 (74.1%) | 7 (100%) | 0.30^b^ |
| Carboplatin + etoposide + durvalumab | 6 (22.2%) | 0 (0%) |  |
| Cisplatin + etoposide + durvalumab | 1 (3.7%) | 0 (0%) |  |

^a^ ECOG-PS 2 versus 0 or 1. ^b^ atezolizumab versus durvalumab. ECOG-PS, Eastern Cooperative Oncology Group performance status; BMI, body mass index; G8, geriatric 8.

B. Treatment after disease progression compared with age (≥75 years versus <75 years)

| Treatment | Age ≥75 years (n =13) | Age <75 years (n=21) |
| --- | --- | --- |
| Best supportive care | 4 (30.8%) | 3 (14.3%) |
| Amrubicin | 8 (61.5%) | 10 (47.6%) |
| Platinum + irinotecan | 0 (0%) | 2 (9.5%) |
| Cisplatin + etoposide | 0 (0%) | 1 (4.8%) |
| Irinotecan | 0 (0%) | 1 (4.8%) |
| Nab-paclitaxel | 1 (7.7%) | 2 (9.5%) |
| Carboplatin + nab-paclitaxel | 0 (0%) | 1 (4.8%) |
| Durvalumab | 0 (0%) | 1 (4.8%) |
